# An early origin for detailed perception in Autism Spectrum Disorder: biased sensitivity for high-spatial frequency information.

**DOI:** 10.1038/srep05475

**Published:** 2014-07-04

**Authors:** Luc Kéïta, Jacalyn Guy, Claude Berthiaume, Laurent Mottron, Armando Bertone

**Affiliations:** 1 Centre de Recherche en Neuropsychologie et Cognition (CERNEC), Université de Montréal; 2 Perceptual Neuroscience Lab for Autism and Development; 3 Integrated Program in Neuroscience, McGill University; 4 Center of Excellence for Pervasive Developmental Disorders (CETEDUM) & Department of Psychiatry, University of Montreal; 5 School/Applied Child Psychology, Department of Education and Counselling Psychology, McGill University

## Abstract

Autistics demonstrate superior performances on several visuo-spatial tasks where local or detailed information processing is advantageous. Altered spatial filtering properties at an early level of visuo-spatial analysis may be a plausible perceptual origin for such detailed perception in Autism Spectrum Disorder. In this study, contrast sensitivity for both luminance and texture-defined vertically-oriented sine-wave gratings were measured across a range of spatial frequencies (0.5, 1, 2, 4 & 8 cpd) for autistics and non-autistic participants. Contrast sensitivity functions and peak frequency ratios were plotted and compared across groups. Results demonstrated that autistic participants were more sensitivity to luminance-defined, high spatial frequency gratings (8 cpd). A group difference in peak distribution was also observed as 35% of autistic participants manifested peak sensitivity for luminance-defined gratings of 4 cpd, compared to only 7% for the comparison group. These findings support that locally-biased perception in Autism Spectrum Disorder originates, at least in part, from differences in response properties of early spatial mechanisms favouring detailed spatial information processing.

Autism spectrum disorder (ASD) is a neurodevelopmental condition characterized by alterations in social communication and interaction, co-occurring with restricted, repetitive patterns of behaviour, interests or activities^1^. It differs from other neurodevelopmental conditions by recurrent demonstrations of superior performances on perceptual and cognitive tasks where local or detailed information processing is advantageous^2^,^3^,^4^,^5^,^6^.

Two neurocognitive theories have been advanced to explain the local bias in autistic perception. The Weak Central Coherence^4^,^7^ (WCC) hypothesis proposes that a decreased influence, or dysfunction, of large-scale neuro-integrative mechanisms results in a reduced global or holistic representation of perceptual information, ultimately leading to a local or detailed processing style. Although the neural basis for such reduced integrative processing in ASD has yet to be elucidated, reduced neural synchrony and/or decreased functional connectivity between cortical areas^8^,^9^,^10^,^11^,^12^ may plausibly underlie large-scale alterations leading to relatively more efficient local analysis.

Alternatively, the Enhanced Perceptual Functioning (EPF) Model^5^,^6^ proposes that an over-functioning of lower-level perceptual mechanisms during the completion of perceptual and cognitive tasks leads to the enhanced extraction of elementary visual and auditory information. A recent functional imaging meta-analysis examining regions involved in cognitive, language and face-processing tasks has supported this view by demonstrating that early visual areas (i.e., striate (BA 17) and extrastriate areas (BA 18, 19)) are activated to a greater degree in individuals with ASD than in those without ASD^13^. These results are in line with the EPF's proposal of a stronger engagement of sensory processing mechanisms in perceptual tasks, including a prominent role of perception in supporting complex cognitive operations.

While both accounts are in agreement with the notion that perception in ASD is locally-oriented and sometimes enhanced, both lack clarity in terms of their underlying neural basis. One reason for this is the fact that the response properties of visual mechanisms responsible for spatial perception in ASD at early levels of processing have not been systematically explored. Studies assessing visual acuity using either clinical screening charts^14^,^15^,^16^ or computer-based paradigms^3^,^17^,^18^,^19^ have for the most part demonstrated that visual acuity is unremarkable in ASD, suggesting that detailed or locally-oriented visual perception in ASD is not of ocular origin. However, it is not ideal to draw firm conclusions on early visuo-spatial processing in ASD based on acuity studies where most target stimuli are considered to be broadband (i.e., Landolt-C optotypes), and do not optimally solicit selective spatial and orientation response properties defining early visual mechanisms^20^. The purpose of the present study was therefore to systematically assess the integrity of neural mechanisms mediating early spatial information processing in ASD by measuring contrast sensitivity functions (CSFs) in a large and well-defined group of autistic participants.

## Methods

### Participants

A total of twenty-one high-functioning autistic (HFA) and fifteen typically developing (TD) adolescents and adults were recruited from the Rivière-des-Prairies Hospital database and participated in the study. Using DSM-IV^21^ criteria, Autistic Disorder (AD) was diagnosed using the algorithm of the Autism Diagnostic Interview-Revised (ADI-R)^22^ combined with the Autistic Diagnostic Observation Schedule-General (ADOS-G)^23^, both conducted by a trained clinician-researcher (LM) who obtained reliability on these instruments. Sixteen of the participants with AD scored above the ADI and ADOS cut-off in the three relevant areas for diagnosis (social, communication, restricted interest and repetitive behaviours). Two participants missed the cut-off score in the ADOS-Communication domain but scored above cut-off on the ADI instrument. Two additional participants received their diagnosis based only on the ADI combined with a non-standardized direct assessment based on the ADOS procedure. One participant was administered an expert clinical DSM-IV diagnosis of AD following a non-standardized direct assessment based on the ADOS procedure. All of the participants with AD had histories of language delay, immediate echolalia, stereotyped language, or pronoun reversal, as assessed by the ADI. All participants with and without AD had a Wechsler Full Scale IQ (FSIQ) greater than 80. Comparison participants and their first-degree relatives were screened with a questionnaire for history of neurological or psychiatric disorders. Autistic and non-autistic participants were matched in terms of Wechsler FSIQ and age (see Table 1). All participants had a normal or corrected-to-normal far and near vision that was assessed before testing using both near and far acuity charts (i.e. near point directional E- and C cards, Snellen letter sequence-A-new Logmar). All participants gave written, informed consent before participating. The study was conducted according to the Declaration of Helsinki and was approved by the ethics committee of Hôpital Rivière-des-Prairies in Montréal, Canada. All participants completed all experimental conditions, which were presented in a counterbalanced manner.

### Apparatus and general procedure

Stimulus generation, presentation and data collection were controlled by a power Macintosh G4 computer. Stimuli were presented on an 18-inch View sonic E90FB .25 CRT monitor (1280 × 1024 pixels), refreshed at a rate of 75 Hz. Generation and animation were controlled by the VPixx (www.vpixx.com) graphics program, which produced a luminance resolution equivalent to an 11-bit video digital-to-analogue converter, resulting in 2048 luminance levels. The mean luminance of the display was 50.0 cd/m^2^ (x = 0.2783, y = 0.3210 in CIE (Commission Internationale de l'Eclairage) u' v' color space) where L_min_ and L_max_ were 0.5 and 99.50 cd/m^2^, respectively. In order to minimize the nonlinearities in the display, the luminance of the monitor was gamma-corrected using a color look-up table. Gamma correction was verified at a regular interval. A Minolta CS-100 Chroma Meter colorimeter was used for the calibration and luminance readings.

### Stimuli

**Figure 1:**
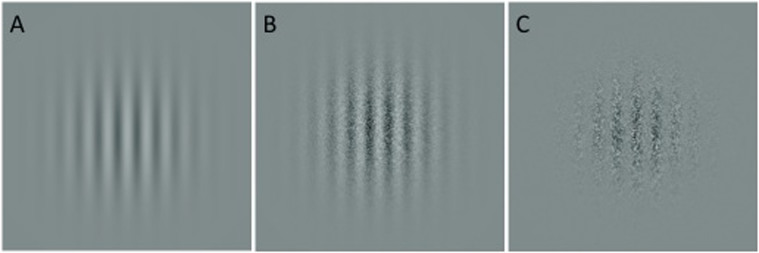
Sample of stimuli used in the present study: vertically-oriented gratings defined by luminance-contrast without (A) and with noise (B), and texture contrast (C).

Contrast sensitivity functions (CSFs) were derived by measuring contrast detection thresholds to vertically-oriented gratings defined by both luminance (with and without noise) and texture contrast (Figure 1). The inclusion of texture-defined stimuli allowed for the assessment of early visual processing beyond the primary visual cortex, which is critical for determining whether increases in stimulus complexity influence sensitivity to detailed information. Stimuli for each condition were constructed by convolving the vertical gratings with spatial frequencies of 0.5, 1, 2, 4, 8 cycles per degree (cpd) with a circular Gaussian envelope (σ = 3.2 deg). The contrast for the luminance (no noise) condition was defined by its Michelson contrast ((L_max_ − L_min_/L_max_ + L_min_) × 100). Contrast detection thresholds to luminance- and texture- defined gratings were also measured, constructed by either adding (luminance contrast) or multiplying (texture contrast) a modulating sinewave to noise pattern consisting of dots (1 pixel × 1 pixel 2 arc min). Individual pixel luminance values for the noise were randomly assigned a value between 24.75 and 74.75 cd/m^24^,^25^.

### Procedure

All experiments were conducted in a dimly lit room where participants comfortably viewed the stimuli from a distance of 57 cm, with head movements minimized using a chinrest. Verbal instructions were given to each participant prior to each experimental block. Practice trials were completed to familiarize the participants with fixation, stimuli presentation and responding. Participants were reminded to fixate on the center of each pattern and encouraged to rest if tired or distracted throughout the testing session. The experimenter remained present throughout testing and initiated successive trials while monitoring fixation and fatigue; no time limit was imposed. Each trial began with the appearance of a small white fixation dot (0.15 deg in diameter) presented centrally on a monitor. A two-interval temporal forced-choice paradigm was then initiated with a press of the spacebar and participants indicated which of two subsequently presented intervals contained the target grating (non-target intervals consisted of uniform grey background). Participant responded by pressing one of two keys on the keyboard. Intervals lasted 500 milliseconds (ms), separated by a 250 ms fixation. Thresholds were obtained for each condition (5 spatial frequency levels × 3 grating attribute types) within a single adaptive staircase (Harvey's ML-PEST) procedure^26^. The staircase used fit a new psychometric function to the data after each trial and ended after a 90% confidence level so that the threshold estimate for all three stimuli fell within 0,1 log units of the true threshold measure. Contrast sensitivity was defined as the inverse of the luminance threshold for the luminance-defined gratings, and the inverse of texture-contrast threshold (texture modulation depth) for the texture defined gratings contrast^24^,^25^. Sensitivity for each attribute condition was then plotted as a function of spatial frequency in order to derive a CSF.

### Data Analysis

The data are presented and analyzed in three different ways for each attribute condition (luminance-defined with and without noise, and texture defined conditions). First, a mixed analysis of variance was conducted on absolute sensitivity (*1/luminance or texture contrast*) using a mixed analysis of variance, with Group as a between subject factor and Spatial Frequency as a within group subject factor. The effect size was estimated by the calculation of *eta* and, in accordance with Cohen^27^, it was considered small if *eta* = *.01*, medium if *eta = .06* and large if *eta = .14*. Second, sensitivity measures were normalized for each participant by dividing the sensitivity of each spatial frequency (Si) by the highest sensitivity (Sh) for each attribute condition (Si/Sh). This was done to reduce the variability to absolute differences in sensitivity between participants, allowing more emphasis to be placed on differential sensitivity to spatial frequency for each participant. Student t-tests were also used to test our *a priori* hypothesis that the AD groups would be more sensitive to high-spatial frequency gratings when defined by luminance^6^,^24^. Finally, categorical data defined by the sensitivity level where participants' highest, or *peak sensitivity* was obtained and examined using a Pearson Chi-square (χ^2^) test for each attribute condition. The data from one autistic participant was excluded from statistical analysis in the luminance-defined with and without noise attribute conditions because thresholds were unattainable on at 2 of the 5 spatial frequencies assessed in each condition.

## Results

Descriptive statistics for age, Wechsler Full Scale Intelligence Quotient (FSIQ), Verbal IQ (VIQ) and Performance IQ (PIQ) for all participants are presented in Table 1. Preliminary analyses were conducted to ensure that FSIQ, VIQ and PIQ were not acting as potential confounding factors. Specifically, we first examined whether significant group differences were present for either VIQ or PIQ. Results from these analyses revealed no significant group differences for any measures of IQ (Table 1). We then examined whether IQ may be correlated with sensitivity across spatial frequency conditions. No significant correlations were found for the AD group. Two significant correlations were found between IQ and sensitivity in the control group. In the first order, no noise condition, sensitivity for 8 cpd was correlated with FSIQ (*r* (15) = .563, *p* = .029) and PIQ *r* (15) = .549, *p* = .029). In the second order, no noise condition, sensitivity for 4 cpd was correlated with, FSIQ (*r* (15) = .566, *p* = .028 and VIQ (*r* (15) = .614, *p* = .015). Given these moderate, yet limited correlations, we found no clear confounding effect of PIQ or VIQ. For this reason, IQ was therefore not used as a covariate during statistical analyses.

### Luminance-defined stimuli (without noise) condition

**Figure 2:**
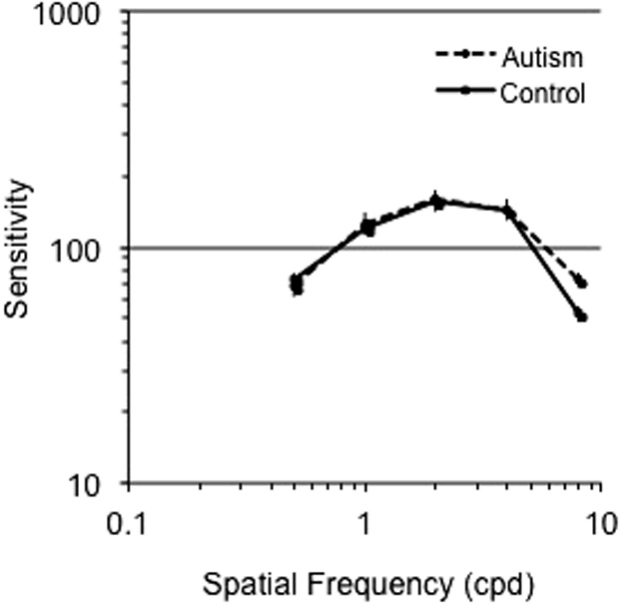
Mean sensitivity for control (solid line) and autism (dashed line) groups as a function of spatial frequency for the luminance, no noise condition.

**Figure 3:**
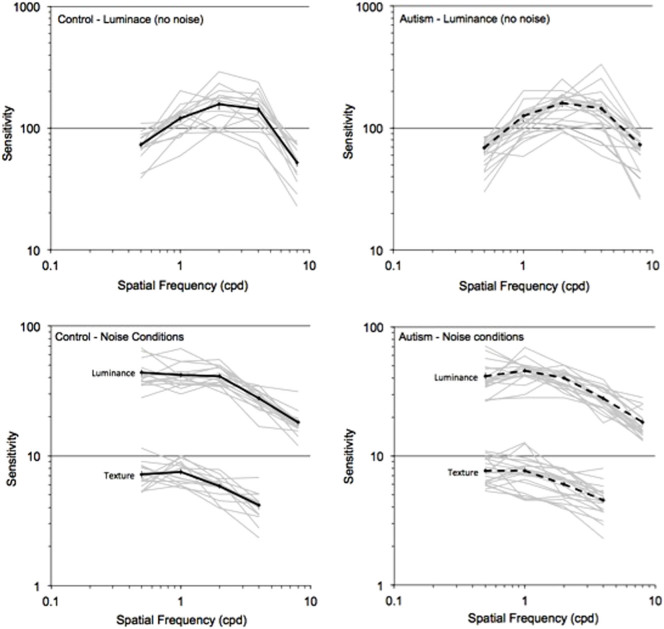
The sensitivity of individual participants in both control (left) and autism (right) groups for the luminance, no noise and noise conditions (bottom) as a function of spatial frequency.

**Figure 4:**
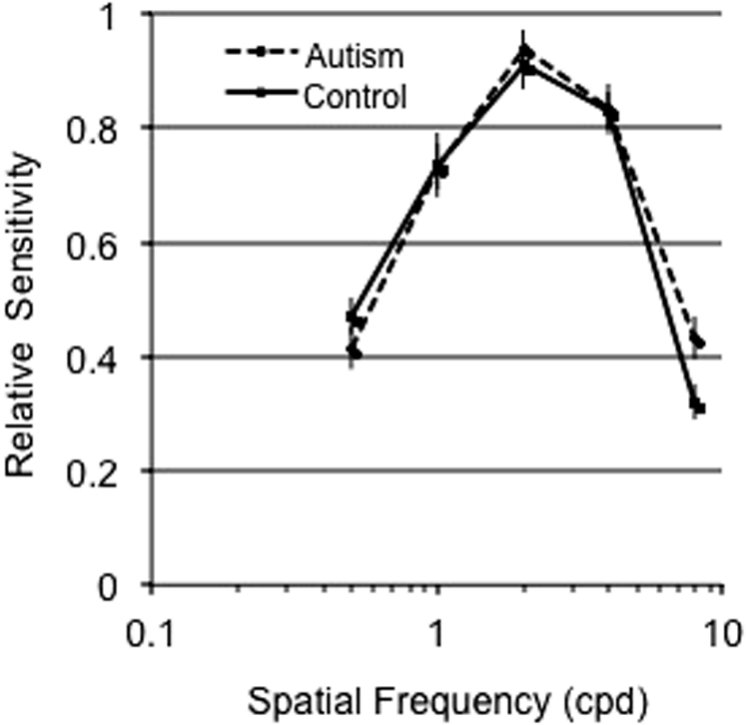
Normalized mean sensitivity measures for control (solid line) and autism groups (dashed line) as a function of spatial frequency for luminance, no noise condition.

Figure 2 shows the mean sensitivity on the y-axis for the control (black line) and AD (dashed line) groups presented as a function of spatial frequencies (SF) plotted on the x-axis. The sensitivity of each individual participant in both control and AD groups is shown in Figure 3. The interaction of Group and Spatial Frequency was significant, *F* (4, 30) = 2.877, *p* = .040, *eta* = .277. We conducted reverse Helmert comparisons to progressively contrast increasing levels of spatial frequencies to their next higher level *(0.5 vs 1 cpd; pooled 0.5 and 1 cpd vs 2 cpd; pooled 0.5, 1 and 2 cpd vs 4 cpd; pooled 0.5, 1, 2 and 4 cpd vs 8 cpd)*. These comparisons revealed that ASD participants had an increased sensitivity for high SFs in comparison to lower SFs (pooled lower frequencies 0.5, 1, 2, 4 cpd) vs highest frequency (8 cpd), *F* (1, 33) = 4.183, p = .049, eta = .112. Moreover, individual contrasts of low and high SFs (i.e., *0.5 cpd vs 8 cpd; 1 cpd vs 8 cpd; 2 cpd vs 8 cpd; 4 cpd vs 8 cpd*) using a conservative Bonferonni corrected alpha level of .0125 (0.05/4) revealed an increased sensitivity in the AD relative to the control group with increasing spatial frequency (*F* (1, 33) = 4.183, p = .007, *eta* = .199). We conducted an hypothesis driven analysis comparing sensitivities of the ASD and TD groups for the highest spatial frequency tested (8 cpd). An independent samples *t*-test revealed a significant effect, *t* (1, 33) = 2.209, *p* = .034, *eta* = .098, indicating a superior sensitivity for the AD group than the control group at a spatial frequency of 8 cpd. We observed the same pattern of results when data were normalized (Figure 4).

**Figure 5:**
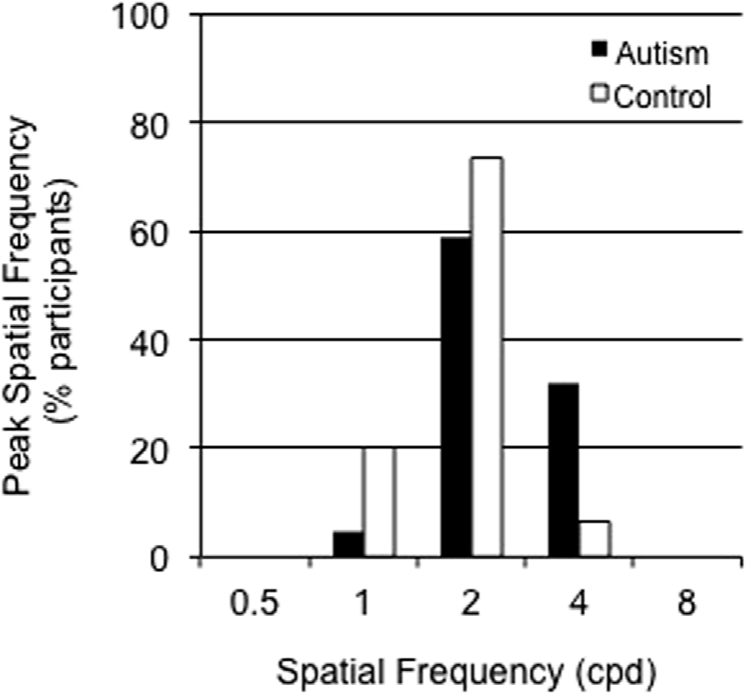
Percentage control (white bars) and autism (black bars) participants with peak sensitivity as a function of spatial frequency for luminance, no noise condition.

A Pearson Chi-square (χ^2^) test was computed to compare peak distribution between our two groups. The results of this analysis revealed a significant between group difference for the peak distribution: χ^2^ (2) = 7.09, *p* = .029 (Figure 5). Specifically, 35% of autistic participants reached peak sensitivity for gratings of 4 cpd compared to 7% in the comparison group. The remaining 93% of participants in the comparison group reached peak sensitivity for gratings of either 1 or 2 cpd.

### Noise conditions

#### Luminance-defined stimuli with noise

The sensitivity of each individual participant in both control and AD groups is shown in Figure 3. A significant Group x Spatial Frequency interaction was not found for this attribute condition, *F* (4, 30) = 1.549, *p* = .214, *eta* = .171. We observed a significant main effect of spatial frequency, *F* (4, 30) = 86.85, *p* < .0001, *eta* = .921. Multiple comparisons contrasting levels of spatial frequencies demonstrated several significant differences: between the 2 cpd and the pooled means of .05 and 1 cpd (*F* (1, 33) = 4.178, *p* = .049, *eta* = .112); between 4 cpd and the pooled mean of the lower frequencies (.05, 1, and 2) (*F* (1, 33) = 148.586, *p* < .0001, *eta* = .818); and finally, between the highest spatial frequency (8 cpd) and the pooled mean of all the lower frequencies (0.5, 1, 2 and 4 cpd), *F* (1, 33) = 4.178, *p* = .049, *eta* = .112. The data analysis conducted with the normalized sensitivity showed the same pattern of results. A Pearson Chi-square (χ^2^) test was conducted to compare peak distribution between the groups. No between group difference was observed for the peak distribution: χ^2^ (2) = .0895, *p* = .639.

#### Texture-defined stimuli (or second-order gratings)

Thresholds for the highest spatial frequency gratings (8 cpd) were not measured for the texture-defined condition, since pilot studies demonstrated that these stimuli were not consistently visible to all participants. The sensitivity of each individual participant in both control and AD groups is shown in Figure 3. Analyses failed to reveal a significant interaction between group and spatial frequency, *F* (3, 32) = .160, *p* = .923, *eta* = .015. Instead, a main effect of spatial frequency was found, *F* (3, 32) = 44.84, *p* < .0001, *eta* = .808. Multiple comparisons contrasting low to high spatial frequencies revealed a significant difference between sensitivity for 2 cpd and the pooled sensitivity to frequencies of 0.5 and 1 cpd, *F* (1, 33) = 39.85, *p* < .0001 .001, *eta* = .555. A significant difference was observed between the 4 cpd and the pooled mean sensitivity for lower frequencies (0.5, 1 and 2 cpd), *F* (1, 33) = 132.513, *p* < .0001, *eta* = .805. A similar pattern of results was observed when data were normalized. A Pearson Chi-square (χ^2^) test was conducted to compare peak distribution between our two groups. No between group differences were observed for the peak distribution: χ^2^ (2) = .264, *p* = .607. In sum, group-differences were not found for conditions where spatial stimuli contained noise, whether luminance- or texture-defined.

## Discussion

The goal of this set of experiments was to measure contrast sensitivity functions (CSFs) for luminance- and texture-defined gratings in a group of autistic participants. A between group difference was found for luminance-defined gratings, with the autistic group demonstrating an increased sensitivity to luminance-defined, high spatial frequency gratings (i.e., 8 cpd). Moreover, there were group differences for the peak distribution of sensitivity for gratings of 4 cpd: 35% of autistic participants reached peak sensitivity at this frequency compared to 7% in the comparison group. Between-group differences in either absolute or peak distribution sensitivity were not found for gratings containing noise, whether defined by either luminance or texture. The former findings are consistent with those of Behrmann et al.^28^ who reported that autistic sensitivity to stimuli of different spatial frequencies (0.13, 0.42, 1.26, 4.19 and 12.6 cpd) defined by luminance modulations embedded in noise was similar to that of a control group. The latter results, however, differ from previous demonstrations of lower sensitivity to static information defined by texture in ASD^24^,^29^. Methodological differences may explain why this occurred as the present investigation examined detection thresholds for sensitivity to vertically-oriented static gratings of varying spatial frequencies, whereas others explored the detection of grating orientations (vertical and horizontal) and object-boundaries with fixed spatial frequency information. The use of noise in our stimuli may moreover have influenced findings related to the texture condition, given that this stimulus attribute it is not best suited to assess mechanisms selective to spatial frequency.

A skewing of peak sensitivity towards higher spatial frequency information in our AD group is consistent with the results of de Jonge et al.^3^, who found a trend toward relatively increased spatial frequency sensitivity for mid- to high-spatial frequencies (6 to 18 cpd) in a group of ASD participants using a Vistech contrast sensitivity chart^30^. More recently, Jemel and colleagues^31^ assessed the contrast sensitivity response properties of early visual-evoked potentials to sine-wave gratings of low, medium and high spatial frequencies in autistic and neurotypical adults matched on IQ. While mid- and high-frequency gratings elicited segregated brain responses in the control group, similar responses to mid and high frequency information were evidenced in the ASD group. They interpret this finding as evidence for altered functional segregation of early spatial filtering mechanisms in ASD, with mid-spatial frequency information being processed by those selective for high-frequency information, ultimately resulting in bias towards detailed visual processing.

In the only previous direct assessment of CSF in ASD, Koh et al.^32^ measured contrast sensitivity functions (CSF) in individuals with and without ASD across a wider range of spatial frequencies. These authors found no group differences on any of the four CSF measures assessed (i.e., visual acuity, peak spatial frequency, peak contrast sensitivity, and contrast sensitivity at a low spatial frequency). However, Koh et al.^32^ used horizontally oriented sinusoidal gratings to derive CSFs, whereas our study used vertically-oriented gratings, the orientation most often used when assessing CSFs in both typical and clinical populations. Second, their study included a rather small (n = 10) and clinically heterogeneous ASD sample including participants diagnosed with autistic disorder (n = 1), Asperger's Syndrome (n = 7) and Pervasive Developmental Disorder - Not Otherwise Specified (n = 2). In contrast, all of the autistic participants in the present study strictly and similarly satisfied DSM IV criteria for Autistic Disorder (AD). This later distinction may underlie the differences between the two studies. Accordingly, speech delay, present in AD and absent in Asperger Syndrome, aggregates with perceptual ability peaks in both visual and auditory domains: speech onset delay predicts higher performance in visuospatial peaks of ability, as measured by the Block Design subtest of the Wechsler intelligence tests^33^. Similarly, within the auditory modality, superior pitch processing (enhanced pitch discrimination for simple tones) is often manifested in participants with a diagnosis of Autistic Disorder, but not Asperger Syndrome^34^,^35^.

Different hypotheses have been advanced to explain why autistics are selectively biased toward detailed visual information. Arguments have focused on various, even complementary alterations of local connectivity within neural assemblies mediating sensory processing, and long-range connectivity between functional brain regions^36^,^37^. Specifically, the locally-oriented and sometimes enhanced autistic perception can be interpreted as reflecting atypical local connectivity affecting the response properties or *tuning* of visual spatial filters^24^,^38^. At a neural level, the response properties of early visual mechanisms responsible for the encoding of elementary visual information, such as orientation and spatial frequency, are modulated by the balance of excitatory/inhibitory activity^39^. Animal and human studies have also demonstrated that GABA mediates this balance in both visual and auditory modalities^40^,^41^. There is also evidence that increased concentrations of GABA in humans are related to lower line orientation^39^ and tactile discrimination thresholds^42^. This supports the hypothesis that GABAergic mechanisms play an important role in sensory discrimination. An alteration of these specific mechanisms may be responsible for increased sensitivity to high-spatial frequency information in ASD^43^, and possibly, in other sensory modalities where GABAergic transmission is involved in shaping the response properties of perceptual mechanisms (i.e., auditory cortex).

Until recently, low-level perception has been overlooked as contributing to ASD's cognitive and behavioural phenotype^36^. Little is known as yet to whether differences in *elementary* perception, exemplified by higher sensitivity to high SFs (or to enhanced pitch perception in the auditory modality) are related to deficits in *social* perception in ASD witht he later are most often interpreted as a reflection of socially-related behaviours^44^. However, some studies have started to assess possible links between elementary and social perception in ASD within a developemntal context^45^. For example, Vlamings et al^46^ demonstrated a processing bias for high-spatial frequency gratings subserving detailed information, concurrent with a detailed-driven approach to facial-expression perception in a group of 3- to 4-year-old children with ASD. These results indicate that an atypical early bias for detailed spatial information in ASD may affect the development of neural mechanisms involved in face processing, with consequences regarding emotion processing and/or social interaction. Local neural alterations mediating perception at the early stages of processing, such as a biased sensitivity to high-spatial frequency information, may therefore be involved in differential autistic performance on higher-level cognitive tasks, whether social in nature or not.

In conclusion, the results of the present study provide a plausible perceptual and biological explanation for superior autistic performance on perceptual tasks that are performed optimally using a detailed-oriented approach. These results are most consistent with *local neural models* of autistic perception^5^,^6^,^43^ that emphasize atypical extraction of *elementary* visual information by neural systems operating within early sensory brain areas^47^,^48^,^49^. Such models are most consistent with ASD's perceptual phenotype being defined by the altered activity of early visual mechanisms, rather than the collateral consequence of a reduced global representation of non-social or social information.

## Author Contributions

L.K. and A.B. designed experiments. L.K., A.B., J.G. and L.M. wrote the main manuscript text. L.K., C.B. and A.B. analyzed the data. L.K. and A.B. prepared Figures 1 through 5 and Table 1. All of the authors (K.L., J.G., C.B., L.M. and A.B.) reviewed the manuscript.

## Figures and Tables

**Table 1:** Characteristics for autistic and control participants

Participant characteristics	Autism	Control	*t* and *p* values
Number	21	15	
Mean age (year)	20.4	19.4	*t* (1, 32.7) = .657, *p* = .516
SD	5.9	3.4	
Range	13–33	14–24	
Mean FISQ	100.26	107	*t* (1, 34) = −.1.39, *p* = .173
SD	13.0	11.6	
Range	81–119	87–122	
Mean VIQ	99.67	108.87	*t* (1, 33.8) = −1.90, *p* = .066
SD	17.1	11.1	
Range	72–124	94–127	
Mean PIQ	103.1	104.2	*t* (1, 34) = −.294, *p* = .771
SD	9.5	12.6	
Range	77–118	82–119	

Wechsler Full Scale Intelligence (FSIQ); Verbal IQ (VIQ); and Performance IQ (PIQ).
